# Pharmaceutical regulations in India: the missing links

**DOI:** 10.1186/1753-6561-6-S5-P9

**Published:** 2012-09-28

**Authors:** Lalitha Narayanan

**Affiliations:** 1 Gujarat Institute of Development Research, Gota, Ahmedabad, India

## Introduction

Modern pharmaceutical industry in India emerged in the 1900s and with that regulation of pharmaceuticals also evolved. The reforms of the 90s while improving the exports, also brought in improved standards of quality to be followed in the entire supply chain of drugs. While for the manufacturers it meant documentation, increased expenditure, and more export opportunities, for the consumer it has resulted in improved drugs, as the public health system also started emphasizing on selecting only those manufacturers with quality standards. However, governance and enforcement mechanisms have to improve to benefit the consumers and the entire healthcare system.

## Methods

Based on a review of literature on pharmaceutical regulations governing manufacturing standards, pharmacy education, and drug prices, this paper discusses the positive outcome of certain regulations and points out where explicit policy focus is needed. Specifically, the paper discusses (a) the positive outcomes of implementing good manufacturing practices (GMP) that has improved the standards of production; (b) the need for aggressive pharmacovigilence practices to enhance awareness on adverse drug reaction; (c) the need to integrate the pharmacy education with the needs of the health systems which functions with far minimal trained human resources particularly in the preventive health care; and (d) the impact of issues related to the drug price control orders on the consumers.

## Results and discussion

Modern pharmaceutical industry in India emerged in the 1900s. Regulation of pharmaceuticals also emerged with the industry. The ultimatum given to the industry to adhere to the schedule M implementation has resulted in improving the standards of medicines produced. While the industry-centric regulations emphasize on quality, little attention is paid particularly to the pharmacovigilence practices to systematically document the adverse drug reactions. The pharmacy education in India has an industry bias. Every year, because of the spate of permissions for pharmacy colleges from the All India Council of Technical Education, pharmacists are churned out in thousands in the country. However, they do not find jobs in the industry. If the pharmacy education is oriented towards community pharmacy or hospital pharmacy, the human resources shortfall in the preventive health care could be improved.

The Drug Price Control Order presently covers only 75 essential drugs and leaves out the new innovator drugs and the biotechnology based drugs, which are prohibitively expensive. While manufacturers wriggle out of price control by producing combinations so that they do not come under the purview of price control, consumers are inundated with irrational an combination that push up the cost of treatment but delays the course of treatment.

The Drugs and Cosmetics Act needs to be revamped taking into consideration the changing industrial scenario. While manufacturers wriggle out of price control by producing combinations so that they do not come under the purview of price control, consumers are inundated with irrational combinations that push up the cost of treatment but delay the course of treatment. Pharmaceutical governance can be vastly improved if the regulatory authorities have more manpower with them.

In India generic drugs are sold in brand names, which push up the cost of treatment as doctors prescribe branded generics. Instead if drugs are sold in generic names, the cost could be contained. The bureaucratic bottlenecks particularly at the state level that delays the spread of *Jan-Aushadhi* stores need to be addressed.

The curriculum of pharmacy education should be revised thoroughly. Pharmacy education in India should include courses like community/hospital pharmacy, where the pharmacist actually assists the doctor in choosing the right medicine after analyzing patients’ case history.

## Competing interests

None declared.

## Funding statement

None declared

